# Microstructure and Bioactivity of Ca- and Mg-Modified Silicon Oxycarbide-Based Amorphous Ceramics

**DOI:** 10.3390/ma17246159

**Published:** 2024-12-17

**Authors:** Qidong Liu, Hongmei Chen, Xiumei Wu, Junjie Yan, Biaobiao Yang, Chenying Shi, Yunping Li, Shu Yu

**Affiliations:** 1 National Key Laboratory of Science and Technology on High-Strength Structural Materials, Central South University, Changsha 410083, Chinawxiumei@csu.edu.cn (X.W.); matti28jy@gmail.com (J.Y.); 2 School of Materials Science and Engineering, Harbin Institute of Technology (Shenzhen), Shenzhen 518055, China; 3 State Key Lab for Powder Metallurgy, Central South University, Changsha 410083, China; biaobiao.yang@imdea.org (B.Y.);; 4 IMDEA Materials Institute, C/Eric Kandel 2, Getafe, 28906 Madrid, Spain; 5 Department of Materials Science, Polytechnic University of Madrid/Universidad Politécnica de Madrid, E.T.S. de Ingenieros de Caminos, 28040 Madrid, Spain; 6 College of Chemistry and Chemical Engineering, Central South University, Changsha 410083, China

**Keywords:** sol–gel, SiOC, network connectivity, apatite forming ability, cytotoxicity

## Abstract

Silicon oxycarbide (SiOC), Ca- and Mg-modified silicon oxycarbide (SiCaOC and SiMgOC) were synthesized via sol–gel processing with subsequent pyrolysis in an inert gas atmosphere. The physicochemical structures of the materials were characterized by XRD, SEM, FTIR, and ^29^Si MAS NMR. Biocompatibility and in vitro bioactivity were detected by MTT, cell adhesion assay, and simulated body fluid (SBF) immersion test. Mg and Ca were successfully doped into the network structure of SiOC, and the non-bridging oxygens (NBO) were formed. The hydroxycarbonate apatite (HCA) was formed on the modified SiOC surface after soaking in simulated body fluid (SBF) for 14 days, and the HCA generation rate of SiCaOC was higher than that of SiMgOC. Accompanying the increase of bioactivity, the network connectivity (NC) of the modified SiOC decreased from 6.05 of SiOC to 5.80 of SiCaOC and 5.60 of SiMgOC. However, structural characterization and biological experiments revealed the nonlinear relationship between the biological activity and NC of the modified SiOC materials.

## 1. Introduction

Bioactive glasses (BGs) have been widely used in bone regeneration and tissue engineering due to their good bioactivity, biocompatibility, excellent chemical stability, and well-defined bonding ability with soft tissues and bone, as well as high bone regeneration capacity [1,2]. The superior bioactivity of BGs is greatly attributed to the formation of the bone-like hydroxycarbonate apatite (HCA, Ca_10_(PO_4_)_6_(OH)_2_·CO_3_) layer in the biological environment, inducing strong bonding of the BG surface to the bone tissue [3]. Rawlings [4] and Strnad [5] proposed the network connectivity (NC) of bioactive glasses and revealed the relationship between NC and bioactivity. To be specific, the tetrahedrons were described by the notation *Q^n^*, in which *n* denoted the number of bridging oxygen atoms in the SiO_4_ unit and varied between zero and four [6]. 

However, two shortcomings limited the application of BGs: the poor mechanical strength and the inability to resist crystallization during the preparation [7,8]. Recently, silicon oxycarbide (SiOC)-based materials have been used in biomedical applications for bone defect repair, such as load-supporting implants or biological coatings [9]. The surface reactivity of SiOC-based materials is similar to that of BGs [10], and they can resist crystallization under higher temperatures (≤1300 °C) [11]. This can be credited to the unique microstructure of SiOC, which has a network of corner-shared silicon-centered tetrahedrons containing both Si–C and Si–O bonds but no C–O bonds [12]. SiOC exhibits the full range of mixed bonded SiO_x_C_4−x_ tetrahedra, i.e., SiO_4_, SiO_3_C, SiO_2_C_2_, SiOC_3_, and SiC_4_, as well as a few free carbons [13,14]. 

Although SiOC exhibits better biocompatibility, it is biologically inert. Doping the SiOC glass network with other elements, especially alkaline earth metals such as Li, Ca, and Mg, can increase biological activity and reduce its NC [15,16]. Calcium (Ca) plays a crucial role in bone formation and resorption as the primary constituent of biological apatite (Ca_10_(PO_4_)_6_(OH)_2_) [17]. Magnesium (Mg) as an abundant and essential mineral in the human body, can inhibit osteoclasts and enhance osteoblast activity [18]. In general, the addition of Ca and Mg was found to enhance the biocompatibility and osteoinduction of the materials and promote cell adhesion [19].For instance, Xie et al. [20] synthesized Ca and/or B-modified SiOC and investigated their crystallization behavior, network architecture, and chemical compositions. Ca/B doping could decrease the NC of SiOC from 5.95 to 5–5.5. However, their investigation was unfortunately limited to the structural evolutions in the material without further delving into the influence of doping on the bioactivity and biocompatibility of SiOC. Chen et al. [21] reported the excellent biocompatibility and bioactivity of SiCaMgOC coatings roughly but showed that they lacked quantitative evaluation of biological activity using advanced techniques as well as a critical understanding regarding the influence of individual elements (Mg or Ca) on the microstructure and bioactivity. Ionescu et al. [22] demonstrated a weak negative correlation between the biological activity and NC of modified SiOC by alkaline earth elements (Mg and Ca), and the quantitative model for glasses could be obtained by calculating the number of bridging and non-bridging oxygens (NBO) per silicon–oxygen tetrahedron in modified SiOC. But, their work in biological activity was only evaluated by immersion in simulated body fluid (SBF) to induce bone-like HAP without incorporating it with other important cell compatibility experiments. 

There are very few reports on the modification research of SiOC-based materials, and there is no systematic correlation between material structure and biological activity. The objective of this study is to examine the bioactivity of Ca- and Mg-modified SiOC and establish the quantifiable evaluation of material bioactivity through the changes of NC. The quantitative structure–bioactivity model through computational algorithms in materials science will also be considered in future work [23], and the highly bioactive SiOC will be applied in bone defect repair.

To this end, the microstructures and bioactivity of Ca- and Mg-modified SiOC amorphous ceramics were investigated thoroughly in comparison with SiOC without any doping. Previous studies demonstrated that with increasing concentrations of Ca and Mg, the bioactivity of the material was enhanced while the biocompatibility decreased. Therefore, we selected the samples with the highest biocompatibility for subsequent experiments. The modified SiOCs doped with Ca and Mg were prepared by the sol–gel method, using dimethyldiethoxysilane and methyltriethoxysilane as precursors and Ca/Mg acetylacetonate as modifiers. The biological activity was evaluated through inducing HCA in SBF at 37 °C. In addition, the biocompatibility of the materials was investigated by MTT assay and cell adhesion assay with mouse fibroblast L929 cells. 

## 2. Materials and Methods

### 2.1. Materials Preparation

To prepare the Ca/Mg modified SiOC glasses, 1 mol dimethyldiethoxysilane (DMDES, AR, 98%, Aladdin, Shanghai, China) and 1 mol methyltriethoxysilane (MTES, AR, 98%, Aladdin, Shanghai, China) were mixed as precursors, which were further mixed with deionized water and anhydrous ethanol to keep the molar ratio of H_2_O/OCH_3_^−^ for 1 and EtOH/Si for 2. After magnetic stirring in a water bath at 40 °C for 30 min, 0.05 mol of Ca acetylacetonate (Ca(acac)_2_, AR, 98%, Aladdin, Shanghai, China) or 0.06 mol of Mg acetylacetonate (Mg(acac)_2_, AR, 98%, Aladdin, Shanghai, China) were added to hydrolysis for 90 min. Note that the Ca:Si molar ratio is 1:40, and Mg:Si molar ratio is 1:33. Hydrochloric acid was added to keep pH at 4. The aged gel can be kept at room temperature for one week. 

C/C substrates (φ 10 × 3 mm, 1.75 g/cm^3^) were manually ground with 400#, 1000#, and 2000# sandpapers, then corroded with aqua regia for 30 min, and cleaned with anhydrous ethanol and deionized water successively. The substrates were coated with aged gel with a lift-off speed of 20 mm/min, then dried at 80 °C, and finally, subjected to pyrolysis in argon gas at 1000 °C to obtain the ceramic coating. The pyrolyzed samples with and without Ca/Mg modified SiOC ceramic coating were named SiCaOC, SiMgOC, and SiOC, respectively. 

### 2.2. Materials Characterization

The phase constitution of the samples was determined by X-ray diffraction (XRD, Advance D8, Bruker, Romanshorn, Switzerland) equipped with Cu-Kα X-ray source in 2*θ* range from 10° to 60° with 6°/min of scanning rate. The surface morphology of samples was observed by SEM (Nova NanoSEM230, FEI, Prague, Czech Republic) in the secondary electron mode with a vacuum degree below 5 × 10^−5^ Pa and an acceleration voltage of 5 kV. The absorption spectra of the materials were recorded through FTIR (Spectrum Two with STA8000, PerkinElmer, Eindhoven, The Netherlands). The microstructures were characterized using ^29^Si MAS NMR. ^29^Si spectra were recorded utilizing Bruker ZG sequence at spinning rates of 8 kHz, pulse angles of 22°, and 120 s relaxation delay. The carbon content of the samples was detected by a CS 600 carbon analyzer (Leco Corporation, St. Joseph, MI, USA), and the oxygen content was detected by a TCH 600 Nitrogen/Oxygen/Hydrogen analyzer (Leco Corporation, MI, USA). In addition, Si, Ca, and Mg contents in SBF were detected by Inductively Coupled Plasma Optical Emission Spectrometry (ICP-OES, TCP-5100-VDV, NYSE: A, Santa Clara, CA, USA).

### 2.3. Mineralization in SBF 

Simulated body fluid (SBF) was prepared according to Kokubo’s [24] method to evaluate the vitro acellular bioactivity. During this experiment, the samples were placed vertically in the SBF at 37 °C. After the immersion, the samples were successively rinsed with acetone (AC, AR, 98%, Aladdin, Shanghai, China), deionized water, and anhydrous ethanol and then dried at 40 °C. The content of release ions was detected by ICP-OES, and the pH of SBF was measured by a pH analyzer (TP110, TIMEPOWER, Shanghai, China), respectively. The microstructures of samples after immersion in SBF were further observed by SEM.

### 2.4. Cytotoxicity Test

MTT (3-[4,5-dimethylthiazol-2-yl]-2,5-diphenyltetrazolium bromide) assay was used to evaluate the L929 cells’ proliferation after in contact with different materials extracts, based on the reduction in tetrazolium salt by living cells [25,26]. To be specific, SiCaOC, SiMgOC, and SiOC were sterilized by the autoclave (LDZM-100KBS, Shanghai, China) and then immersed in the complete medium for 24 h, 48 h, and 72 h to obtain extracts of different concentrations. Materials extracts and Dulcecco’s modified eagle medium (DMEM) were used as experimental groups and the negative control group, respectively.

L929 cells were cultured in DMEM supplemented with 10% fetal calf serum (FCS) at 37 °C and 5% CO_2_. Then, 100 μL cell suspension with a density of 5 × 10^4^ cells/mL was seeded onto the bottom of the 96-well cell culture plate and continuously cultured for 24 h. After 24 h of cell culture, 100 μL material extract or 100 μL DMEM was added to cell suspension corresponding to the experimental groups and negative control group, respectively. The cells were incubated at 37 °C and 5% CO_2_ for 72 h. After that, 10 μL of 5 mg/mL MTT was added to all groups. The cells were incubated in the mixture of MTT and DMEM for 4 h. After that, 150 μL dimethyl sulfoxide (DMSO) was added to dissolve the cells. The optical absorbance (OD) of the supernatant was measured at a wavelength of 630 nm using the automatic plate reader (ELX800, BioTek, Winooski, VT, USA). The relative growth rate (RGR) of the cells was determined according to the formula in Equation (1) [27]:

(1) $$\mathrm{RGR}\left(\%\right)=\frac{\mathrm{OD}\left(\mathrm{experimental}\;\mathrm{group}\right)}{\mathrm{OD}\left(\mathrm{negative}\;\mathrm{control}\right)}\times 100\%$$

Cell viability (RGR) of the negative control group measured by the MTT method was defined as 100%. Then, the RGR of the experimental groups was calculated relative to the negative control group. The cytotoxicity of the material is categorized into different grades: RGR ≥ 100%; 75~95%; and 50~74% for grades 0, 1, and 2, respectively. Both grades 0 and 1 stand for non-cytotoxicity. Moreover, grade 2 corresponds to non-cytotoxicity or cytotoxicity, depending on the final morphology of cells. The experiment was duplicated three times under the same conditions.

### 2.5. Cell Adhesion Assay

L929 cell suspension with a density of 5 × 10^4^ cells/mL was dropped evenly on the surface of the three samples. After 72 h incubation, the samples were rinsed with phosphate buffer saline (PBS), then fixed with 2.5% glutaraldehyde solution for 2 h, and finally dehydrated with ethanol. The morphology of the cells adhered to the surface of the materials was further observed by SEM.

### 2.6. Statistical Analysis 

All the analyses were repeated at least three times, and the data were analyzed by GraphPad Prism (ver.9.0.0) software using ANOVA one and two-way followed by Tukey post hoc test to determine the difference significance [28,29]. The significances were shown by *p*-value using * sign.

## 3. Results and Discussion

### 3.1. Microstructures

**Figure 1:**
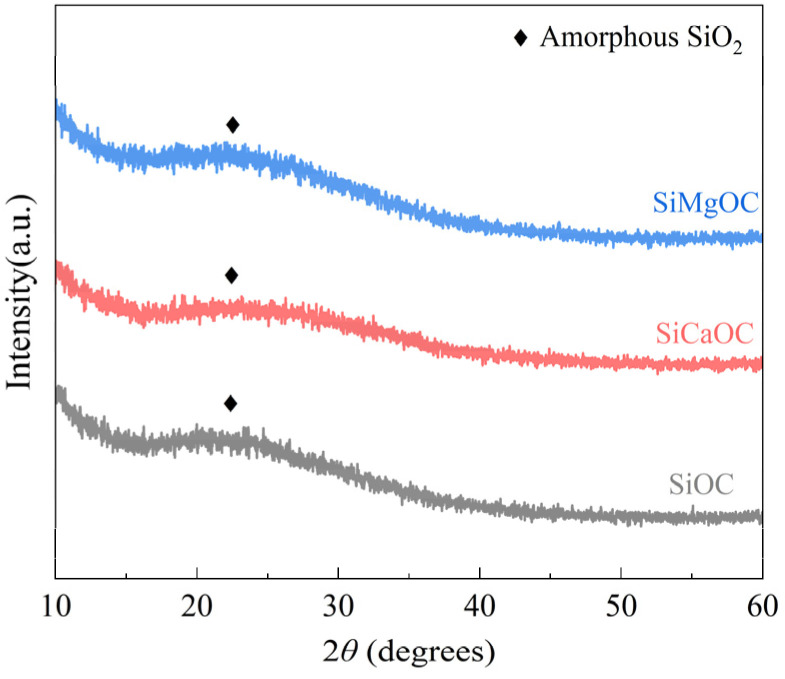
XRD analysis of different samples after 1000 °C heat treatment.

The phase constitutions of Ca-modified and Mg-modified SiOC glasses, as well as SiOC without any doping, were analyzed by XRD, as shown in Figure 1. The incorporation of Ca or Mg did not change the phase constitution of SiOC, i.e., amorphous SiO_2_ corresponding to a broad peak between 20° and 25°, which indicated that the three materials were completely amorphous after 1000 °C pyrolysis.

**Figure 2:**
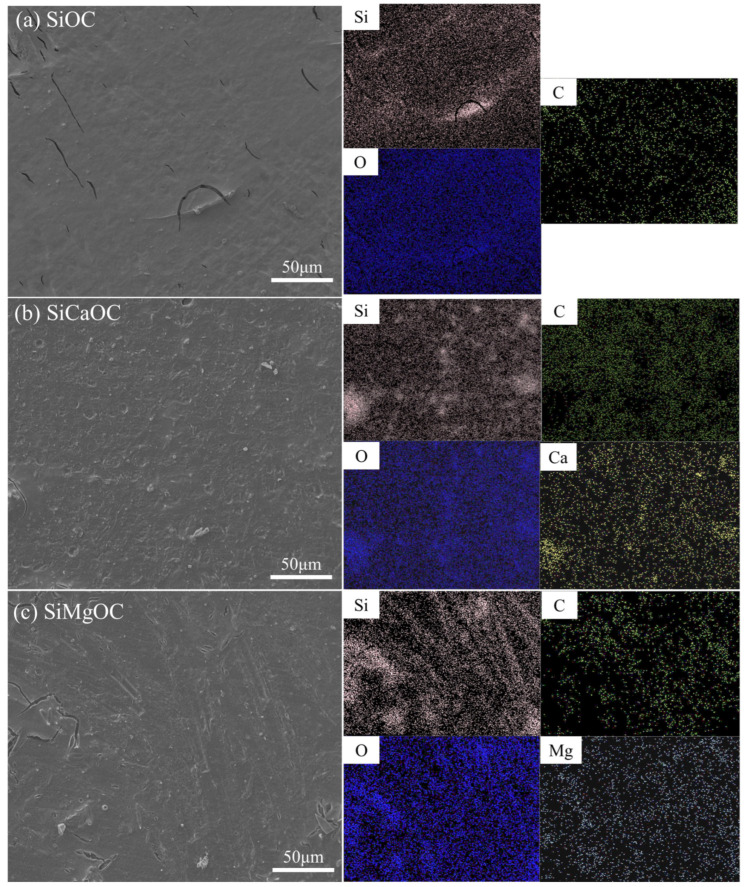
SEM and EDS analysis of different materials: (**a**) SiOC glass; (**b**) SiCaOC glass; (**c**) SiMgOC glass.

Surface morphology and elemental distribution of initial and modified SiOC are shown in Figure 2. The surface of SiOC was relatively smooth, with some minor cracks caused by thermal stress during the pyrolysis process. In comparison with SiOC, the SiCaOC had a much rougher surface with some obvious puddles, while the SiMgOC displayed a rougher surface with some pronounced cracks. SiCaOC and SiMgOC showed fewer cracks, maybe because the rougher surfaces were in favor of stress release. Further EDS results evidenced that both Ca and Mg have been distributed uniformly on the surface of glass materials.

**Figure 3:**
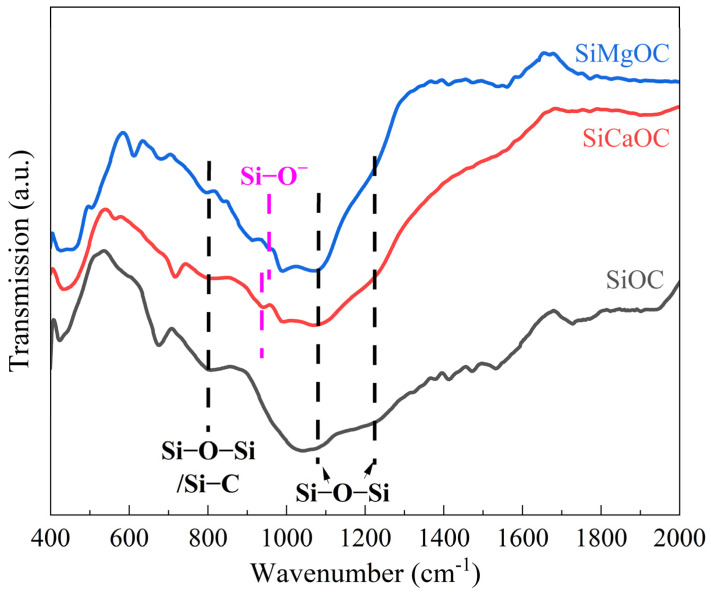
FTIR spectra of SiOC, SiCaOC, and SiMgOC glasses.

The chemical bands of three glass samples were investigated by FTIR spectroscopy, as shown in Figure 3. Both the absorptions at around 1083 and 1220 cm^−1^ correspond to the asymmetric stretching vibrations of the Si–O–Si [20,30]. The absorption at 800 cm^−1^ was overlapped by symmetric stretching and bending vibration of Si–O–Si, as well as bending vibration of Si–C. According to a study by Lyu et al. [31], the modification of Ca and Hf into the SiOC can form silica sites with NBO (Si–O^−^), leading to the appearance of absorption in the range of 900–970 cm^−1^. In this manner, the new absorptions appearing at 939 cm^−1^ and 957 cm^−1^ could be explained by the presence of NBO in SiO_4_ tetrahedral units [32]. This also indicates the successful fabrication of SiCaOC and SiMgOC. 

**Figure 4:**
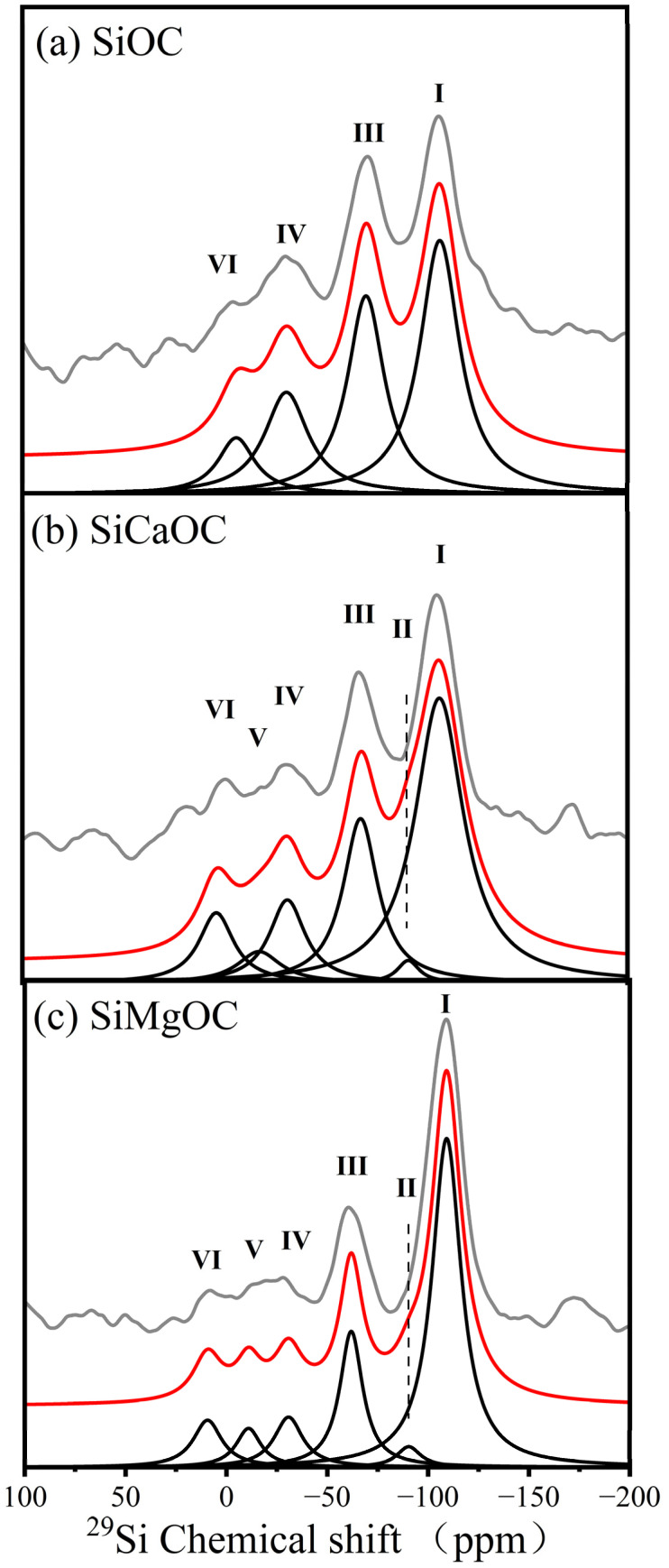
^29^Si MAS NMR of three materials: (**a**) SiOC glass; (**b**) SiCaOC glass; (**c**) SiMgOC glass. The experimental (grey line) and simulated (red line) spectra, as well as the individual simulation components (black lines), are shown. The results of the simulation correspond to Q_4_SiO_4_ (I), Q_3_SiO_4_ (II), SiO_3_C (III), SiO_2_C_2_ (IV), SiOC_3_ (V), and SiC_4_ (VI).

The microstructure and network architecture of samples were investigated by ^29^Si MAS NMR spectra (Figure 4). In addition, based on the experimental NMR data, the values of NC for various glass samples were determined from the following equation [22]:

(2) $$\mathrm{NC}=\left[4\times f\left(Q^{4}\right)\right]+\left[3\times f\left(Q^{3}\right)\right]+\left[2\times f\left(Q^{2}\right)\right]+\left[1\times f\left(Q^{1}\right)\right]+\left[6\times f\left(\mathrm{Si}O_{3}C\right)\right]+\;\left[8\times f\left(\mathrm{Si}O_{2}C_{2}\right)\right]+\left[10\times f\left(\mathrm{SiO}C_{3}\right)\right]+\left[12\times f\left(\mathrm{Si}C_{4}\right)\right]$$

with *Q* being the Si atomic fractions among total network formers and $f\left(x\right)$ the site fraction of the corresponding species *x* from the experimental NMR data. The NC^f^ of the samples was calculated by Equation (2) and summarized in Table 1.

The spectra of SiOC in Figure 4a were fitted with Gaussian peaks at around −107, −71, −34, and −7 ppm, which correspond to the Q_4_SiO_4_(I), SiO_3_C (III), SiO_2_C_2_ (IV), and SiC_4_ (VI), respectively [12]. Furthermore, two new peaks at −91 and −11 ppm of both SiCaOC and SiMgOC corresponded with Q_3_SiO_4_ (II) and SiOC_3_ (V), respectively, and those peaks were hardly observed in the spectra of SiOC (Figure 4). 

The relative fractions of different silicon species in three studied glass materials that were analyzed from NMR spectra are shown in Table 1. Additionally, the relative fractions of various SiOC-based glasses were also determined by carbon analyzer, nitrogen/oxygen/hydrogen analyzer, and ICP-OES, whose results were roughly in line with the NMR composition except the higher content of carbon for the free carbon existed in SiOC glass, rather than in the SiOC network (Table 1). 

The proportion of Q_4_SiO_4_ sites increased significantly from 42.1% for SiOC to 51.6% for SiCaOC and 56.8% for SiMgOC (Table 1). Accordingly, the fractions of SiO_3_C, SiO_2_C_2_, SiOC_3_, and SiC_4_ sites decreased after modification. Moreover, the addition of alkaline earth metals results in the formation of a small fraction of Q_3_SiO_4_ sites, i.e., 1.8% for SiCaOC and 2.8% for SiMgOC. The NMR composition showed that the O/Si ratio increased from 1.49 for SiOC to 1.54 for SiCaOC and 1.59 for SiMgOC, while the C/Si ratio decreased from 0.25 for SiOC to 0.23 for SiCaOC and 0.20 for SiMgOC. That means more oxygen-rich Si tetrahedral was formed after doping of Ca or Mg. Moreover, the modification with Ca or Mg reduced the NC of the SiOC-based glasses, from 6.05 for SiOC to 5.80 for SiCaOC and 5.60 for SiMgOC (Table 1).

### 3.2. In Vitro Bioactivity

**Figure 5:**
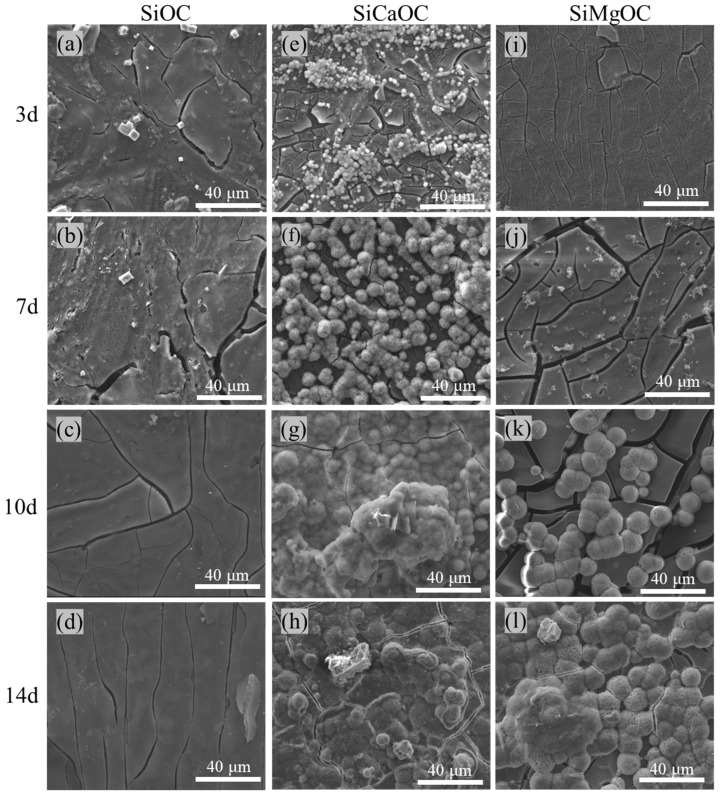
The surface morphology of the samples immersed in SBF for different immersion times: (**a**–**d**) SiOC glass; (**e**–**h**) SiCaOC glass; (**i**–**l**) SiMgOC glass.

The apatite-forming ability of SiOC, SiCaOC, and SiMgOC were evaluated by immersion in SBF, and their surface morphologies after different immersion periods were observed by SEM, as shown in Figure 5. The surface of SiOC remained unchanged even after the immersion in SBF for 14 days (Figure 5a–d). However, clear spherical sediments with a diameter of 1–3 μm were observed on the surface of SiCaOC after immersion in SBF for 3 days (Figure 5e). As the immersion time prolonged to 7 days, these sediments were observed to grow up with a diameter of 6–10 μm (Figure 5f). At an immersion time longer than 10 days, the surface of SiCaOC was coated fully with these sediments (Figure 5g–h). The surface evolution of SiMgOC in SBF was similar to that of SiCaOC but had two differences. The first one was that the appearance of sediments in SiMgOC seemed to be postponed since no sediments formed at 3 days of immersion (Figure 5i) and limited sediments appeared at 7 days of immersion (Figure 5j) with almost full coverage of sediments until 14 days of immersion (Figure 5l). This demonstrates that SiCaOC has a higher ability to generate sediments than SiMgOC. The other difference is in the larger size of sediments in SiMgOC (diameter: 5–15 μm) compared to that of SiCaOC. 

**Figure 6:**
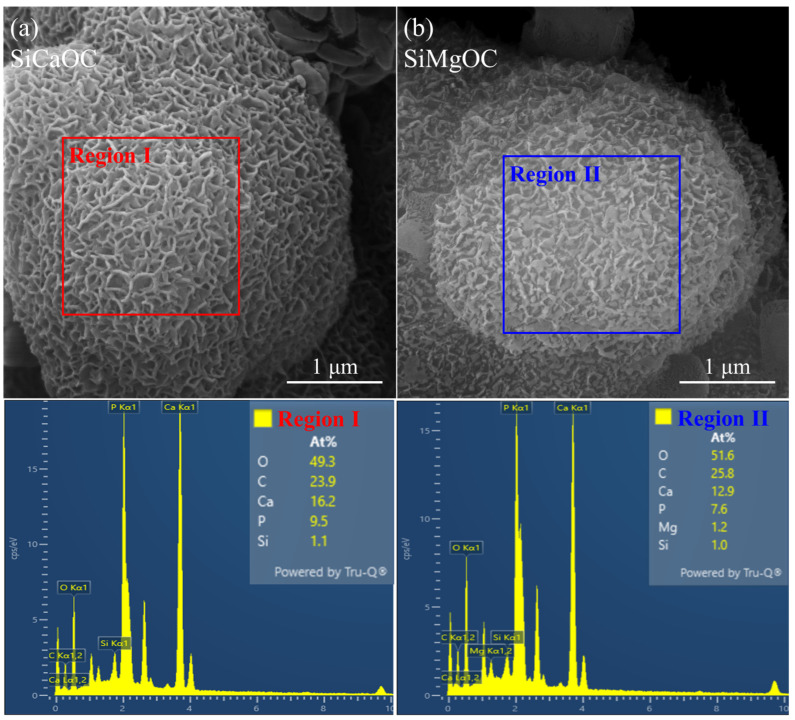
SEM morphology and corresponding EDS spectra of glass materials after 14-day immersion in SBF: (**a**) SiCaOC and (**b**) SiMgOC.

The morphologies of sediments at higher magnifications and corresponding EDS analysis for SiCaOC and SiMgOC are shown in Figure 6. Both sediments exhibit a spherical cauliflower morphology, which is consistent with previous studies by Haider et al. [33] and Chen et al. [34]. Further EDS analysis revealed that the Ca/P molar ratio of the sediments was 1.70 for SiCaOC and 1.69 for SiMgOC, respectively. Note that both values were almost identical to that of stoichiometric HCA (1.69) [35], whose appearance can also be evidenced by the formation of crystal nuclei in epitaxy on the material surface (Figure 6) [36]. The modified SiOC (SiCaOC or SiMgOC) showed a higher bioactivity than SiOC, given they can form HCA in SBF, and the induction of HCA for SiCaOC was faster than that of SiMgOC. This may be related to the fact that Ca is the necessary element of HCA as well as Mg^2+^, which can reduce the conversion rate of amorphous calcium–phosphate to the crystalline HCA [37,38]. 

**Figure 7:**
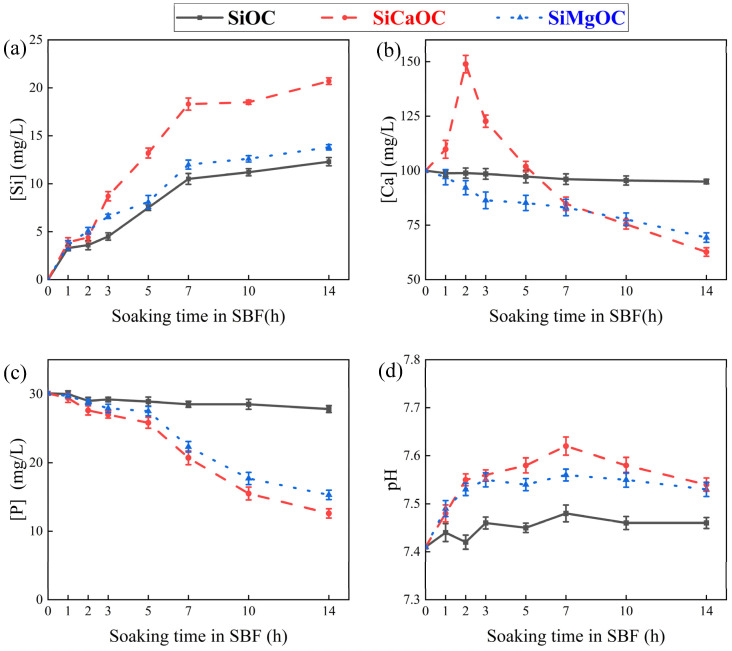
The change in different ions content and pH of SBF after the immersion tests for various glass materials: (**a**) [Si]; (**b**) [Ca]; (**c**) [P]; and (**d**) pH.

Figure 7 shows the change in different ion concentrations and pH values of the SBF for 14 days. The Si ion concentration of all materials gradually increased as immersion time increased, while the releasing rate of Si was sensitive to the materials, i.e., SiCaOC > SiMgOC > SiOC (Figure 7a). However, the evolution of Ca ion concentration with respect to the immersion time depends on the material itself. Ca ion of SiCaOC increased sharply to 148.9 mg/L in the first two days for the release of Ca^2+^ from SiCaOC, then decreased drastically to the lowest level (62.7 mg/L) after 14 days among all the materials (Figure 7b). With the immersion time increased, SiMgOC exhibited a continuous decline in Ca ion concentration, reaching the value of 69.3 mg/L after 14 days. A similar decreasing trend was observed for SiOC, while this decreasing rate was much lower in comparison with SiMgOC (Figure 7b). The concentration of P ions in the SBF of all experimental groups exhibited a gradual decrease with increasing immersion time, and the decreasing rate was sensitive to the materials, i.e., SiCaOC > SiMgOC > SiOC (~0) (Figure 7c). Note that the generation of HCA in SBF may explain the decline in Ca and P ion concentrations of SBF during the immersion of SiCaOC and SiMgOC. The pH of SBF immersed by both SiCaOC and SiMgOC increased rapidly but then remained almost constant as immersion time increased, but both were higher than that of SiOC (Figure 7d). The increase in pH for SiCaOC and SiMgOC after initial immersion could be related to the exchange of Ca^2+^ or Mg^2+^ in the materials with H^+^ or H_3_O^+^ in the SBF, accompanied by the formation of Si–OH and the breaking of Si–O–Si in materials [39,40]. The subsequent formation of the HCA layer consumed carbonate and phosphate ions, leading to the formation of more H^+^ or H_3_O^+^ in the solution via shifting the following reversible reactions in the right direction [41]:

(3) $${\mathrm{HCO}}_{3}^{-}↔{\mathrm{CO}}_{3}^{2+}+H^{+}$$

(4) $${\mathrm{HPO}}_{4}^{2-}↔{\mathrm{PO}}_{4}^{3-}+H^{+}$$

Their combination brought the constant value of pH for SBF immersed by SiCaOC and SiMgOC, while the missing of these reactions in SiOC should be responsible for the stable value of pH (Figure 5). In addition, after 14 days, the pH reached ~7.54 for SiCaOC and SiMgOC, slightly higher than that for SiOC (~7.45). 

### 3.3. Biocompatibility Assessment

**Figure 8:**
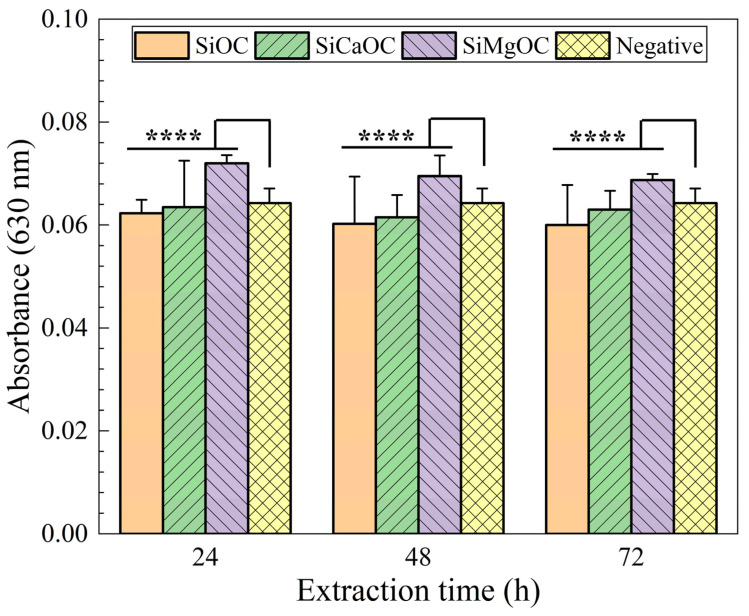
Absorbance of the different samples in MTT toxicity test. n = 3, for each group (**** *p* < 0.0001).

The cytotoxicity of the studied glass materials was evaluated by MTT assay with L929 cells for culturing for 3 days, with the results shown in Figure 8 and Table 2. With the increase in extraction time, the absorbance of all experimental groups decreased, indicating the number of living cells decreased. The cell viability of SiMgOC was higher than that of the negative control, while the other two experimental groups (SiCaOC and SiOC) were lower than that of the negative control (Figure 8). In addition, the cytotoxicity grade of SiMgOC was 0, while that of SiOC and SiCaOC was 1, which indicated that SiMgOC was the least toxic to cells, followed by SiCaOC and SiOC. It was noteworthy that the cytotoxicity grade of either 0 or 1 indicated non-cytotoxicity [27]. Furthermore, SiMgOC extract can significantly promote cell reproduction and differentiation, especially in low concentrations. The results of the cytotoxicity test were in agreement with those previously reported by Chen et al. [21].

**Figure 9:**
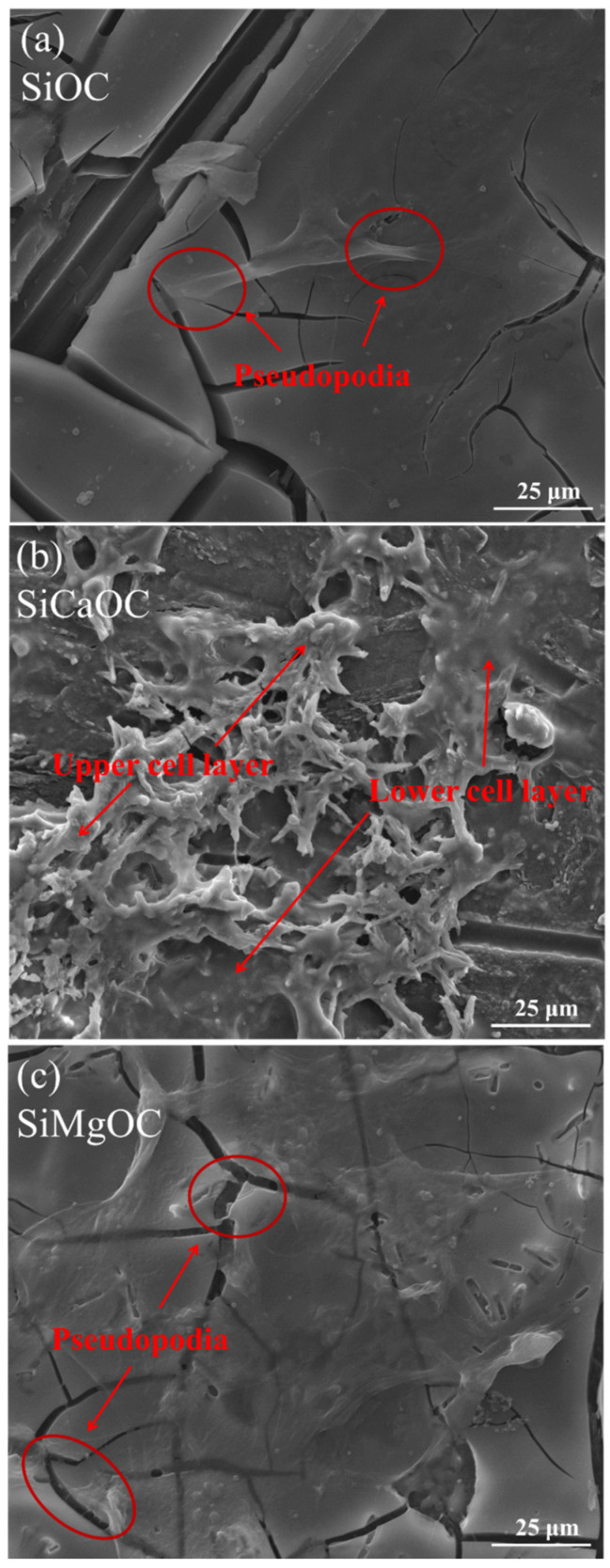
SEM images of L929 cells attached to the surface of the different samples: (**a**) SiOC; (**b**) SiCaOC; and (**c**) SiMgOC.

Figure 9 shows the L929 cell morphology on the surface of the materials with the same culture time of 72 h. The densest cells were observed on the surface of SiCaOC, followed by SiMgOC and SiOC, accompanied by the decreasing bioactivity in Figure 5. Few cells attached to the surface of SiOC grew well with obvious pseudopods (Figure 9a). On the surface of SiCaOC, at least two layers of cells were observed (Figure 9b), causing poor growth or death of cells. However, the surface of the SiMgOC was covered with a layer of cells, and the cells grew well even around the cracks of the surface (Figure 9c). This could explain well why the cytotoxicity of SiCaOC was higher than that of SiMgOC (Table 2), although the bioactivity of SiCaOC was obviously higher than that of SiMgOC (Figure 5).

### 3.4. Correlation Between Microstructure and Bioactivity

**Figure 10:**
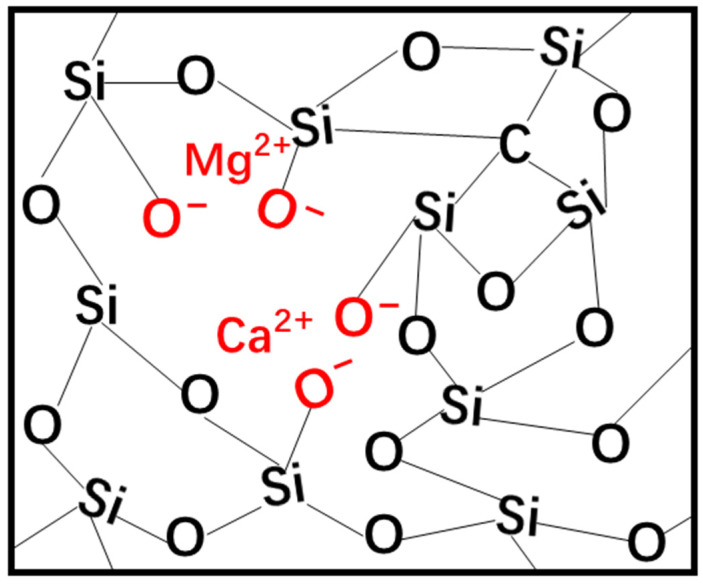
Schematic illustration of the doping of Ca and Mg into the SiOC network structure.

The doping of Ca and Mg resulted in the breakdown of the Si–O–Si bonds in the SiOC network structure, leading to the formation of NBO (Si–O^−^), as schematically shown in Figure 10. The Si–O^−^ does not connect to the other ions directly by “bonding”, but rather, it interacts with Ca^2+^ or Mg^2+^ through electrostatic interactions [9,15]. The formation of NBO results in the disruption of the glass network, which, in turn, renders the glass more reactive in aqueous solution like SBF (Figure 5). 

According to the research of Shearer et al. [19], the apatite formation of glass was dependent on the level of polymerization. The higher the NC of the glass, the lower the ion concentration released, which slowed down the formation of apatite. The formation of NBO (Figure 3 and Figure 4) decreased the NC (Table 1) and, thus, improved the glass bioactivity in SBF (Figure 5). However, the NC of SiMgOC was lower than that of SiCaOC, but SiCaOC showed higher bioactivity, as evidenced by faster induction of HCA on the surface (Figure 5). This suggested that the NC may not be the sole determinant of the bioactivity of glass materials, and other microstructural features should be included in designing SiOC-based glasses with excellent bioactivity. In addition, the higher bioactivity still cannot guarantee lower cytotoxicity since multiple layers of cells may be formed on the surface, leading to the constrained growth and premature death of cells (Figure 9). These complicated scenarios for the selection of doping elements should be carefully treated after involving more microstructural factors. The above results demonstrated that the modified SiOC exhibited excellent bioactivity and biocompatibility, thereby indicating its potential for application in the biomedical field of bone defect repair.

## 4. Conclusions

In this study, SiOC, SiCaOC, and SiCaOC were successfully synthesized by sol–gel method. The microstructure and bioactivity of these three glasses were observed and quantitatively evaluated, respectively. The following conclusions can be derived from this study:

(1) Ca and Mg were successfully doped into the network structure of SiOC by breaking down the Si–O–Si bonding, leading to the formation of NBO. The incorporation of Ca and Mg significantly reduced the NC of SiOC glasses from 6.05 to 5.80 of SiCaOC and 5.60 of SiMgOC, respectively;
(2) The bioactivity of modified materials was obviously improved, as evidenced by the more rapid deposition of HCA on SiCaOC and SiMgOC. Both SiCaOC and SiMgOC demonstrated excellent biocompatibility, with the cells exhibiting distinct growth on their surface;
(3) Among the three glasses, the highest bioactivity was found in SiCaOC, while the lowest cytotoxicity was found in SiMgOC. Excessive bioactivity will lead to multiple layers of cells on the surface, causing constrained growth as well as premature death of cells;
(4) NC was not the sole determinant of the materials’ bioactivity, and other microstructural features should be included in designing high-performance SiOC-based glasses;
(5) The biocompatibility experiments conducted in this study are preliminary and insufficient. Comprehensive evaluations, including cell proliferation, sensitization, and teratogenicity, as well as animal implantation, should be conducted in our next research.

## Figures and Tables

**Table 1:** Silicon site fractions of three SiOC-based glasses determined from ^29^Si–MAS NMR spectra.

Sample	Q^4^SiO_4_ (I)	Q^3^SiO_4_ (II)	SiO_3_C (III)	SiO_2_C_2_ (IV)	SiOC_3_ (V)	SiC_4_ (VI)	NMR Composition	Measured Composition	NC
SiOC	42.1%	_	30.6%	18.6%	_	8.7%	Si_1_O_1.49_C_0.25_	Si_1_O_1.49_C_0.51_	6.05
SiCaOC	51.6%	1.8%	21.3%	11.1%	9.2%	5.0%	Si_1_Ca_0.05_O_1.54_C_0.23_	Si_1_Ca_0.05_O_1.54_C_0.56_	5.80
SiMgOC	56.8%	2.8%	18.4%	8.4%	7.9%	5.7%	Si_1_Mg_0.06_O_1.59_C_0.20_	Si_1_Mg_0.06_O_1.59_C_0.57_	5.60

**Table 2:** The cytotoxicity grades of the materials.

Samples	Grade		
	Extraction of 24 h	Extraction of 48 h	Extraction of 72 h
SiOC	1	1	1
SiCaOC	1	1	1
SiMgOC	0	0	0
Negative	0	0	0

## Data Availability

The original contributions presented in the study are included in the article, and further inquiries can be directed to the corresponding author.
